# Quinoa Protein/Sodium Alginate Complex-Stabilized Pickering Emulsion for Sustained Release of Curcumin and Enhanced Anticancer Activity Against HeLa Cells

**DOI:** 10.3390/foods14152705

**Published:** 2025-08-01

**Authors:** Yiqun Zhu, Jianan Li, Shuhong Liu, Hongli Yang, Fei Lu, Minpeng Zhu

**Affiliations:** College of Grain Science and Technology, Shenyang Normal University, Shenyang 110034, China; zyq06101917@163.com (Y.Z.); 18334964397@163.com (J.L.); liushuhong1230@163.com (S.L.); yanghongli1982@163.com (H.Y.)

**Keywords:** quinoa protein isolate, Pickering emulsion, curcumin delivery, controlled release, cytotoxicity, bioaccessibility

## Abstract

Quinoa protein isolate (QPI) and sodium alginate (SA) have excellent biocompatibility and functional properties, making them promising candidates for food-grade delivery systems. In this study, we developed, for the first time, a QPI/SA complex-stabilized Pickering emulsion for curcumin encapsulation. The coacervation behavior of QPI and SA was investigated from pH 1.6 to 7.5, and the structural and interfacial characteristics of the complexes were analyzed using zeta potential measurements, Fourier-transform infrared spectroscopy, scanning electron microscopy, and contact angle analysis. The results showed that the formation of QPI/SA complexes was primarily driven by electrostatic interactions, hydrogen bonding, and hydrophobic interactions, with enhanced amphiphilicity observed under optimal conditions (QPI/SA = 5:1, pH 5). The QPI/SA-stabilized Pickering emulsions demonstrated excellent emulsification performance and storage stability, maintaining an emulsification index above 90% after 7 d when prepared with 60% oil phase. In vitro digestion studies revealed stage-specific curcumin release, with sustained release in simulated gastric fluid (21.13%) and enhanced release in intestinal fluid (88.21%). Cytotoxicity assays using HeLa cells confirmed the biocompatibility of QPI/SA complexes (≤500 μg/mL), while curcumin-loaded emulsions exhibited dose-dependent anticancer activity. These findings suggest that QPI/SA holds significant potential for applications in functional foods and oral delivery systems.

## 1. Introduction

Pickering emulsions are surfactant-free emulsions stabilized by solid colloidal particles that irreversibly adsorb at the oil–water interface, forming a rigid interfacial layer [1,2]. This particle-based stabilization mechanism provides emulsions with exceptional stability against droplet coalescence, creaming, and environmental stresses, outperforming conventional emulsions [3]. Because of these advantages, Pickering emulsions have attracted growing interest across various fields, including food, pharmaceuticals, cosmetics, and functional materials [4,5]. However, traditional stabilizers such as silica, clay, calcium carbonate, and magnetic nanoparticles present challenges related to biocompatibility, biodegradability, and delivery efficiency, particularly in food and biomedical applications [6]. Consequently, the use of natural macromolecules—especially proteins and polysaccharides—as food-grade and biocompatible alternatives has emerged as a promising direction [7,8]. Although various plant- and animal-derived proteins have been explored for this purpose, their poor solubility, hydrophobicity, and conformational instability often result in emulsion destabilization phenomena such as creaming, flocculation, and aggregation [9]. To improve protein-based emulsification, modification techniques or synergistic formulations with polysaccharides have been proposed [10]. Polysaccharides, typically hydrophilic and anionic, can interact with proteins to improve emulsions’ stability [11,12]. Although covalent modifications (e.g., glycosylation, acylation, esterification) can strengthen these interactions, they often require complex procedures and may generate undesirable byproducts [13]. In contrast, non-covalent interactions—dominated by electrostatic attraction, hydrophobic interactions, hydrogen bonding, and van der Waals forces—offer a milder and safer approach to protein–polysaccharide complex formation [14,15]. This approach enhances protein hydrophilicity and interfacial activity while avoiding chemical crosslinkers, making it ideal for developing safe, sustainable, and clean-label Pickering emulsions [2].

Quinoa protein (QPI), derived from *Chenopodium quinoa*, is widely regarded as a high-quality, complete plant-based protein source [16]. Unlike most plant proteins, QPI contains all nine essential amino acids required by the human body, is naturally gluten-free, and exhibits high protein content and a high amino acid score, making it a promising alternative to animal proteins and a potential future staple in sustainable food systems [16]. QPI has been extensively applied in the food industry and has demonstrated excellent nutritional functionality [17,18]. Moreover, emerging research indicates that QPI possesses potential prebiotic effects, including the regulation of the intestinal microbiota and support of gastrointestinal health [19,20,21]. Given these nutritional and functional advantages, QPI holds significant promise for application in novel food formulations and bioactive delivery systems.

Sodium alginate (SA) is a natural anionic polysaccharide typically extracted from brown algae or kelp [22,23]. It is widely used in food production as a stabilizer and thickener due to its excellent biocompatibility, safety, and gel-forming properties [24,25,26]. Structurally, SA contains abundant carboxyl and hydroxyl groups that enable it to interact with polycations, such as proteins, through electrostatic attraction and hydrogen bonding [27,28]. These interactions allow SA to form complexes with various proteins—such as whey protein isolate (WPI), zein, and myofibrillar proteins—which have been successfully applied in emulsion stabilization [29,30]. For example, SA/WPI complexes have been shown to enhance the thermal and physicochemical stability of β-carotene-loaded emulsions [31], while SA/zein complexes improve the antioxidant capacity and stability of Pickering emulsions [32]. Similarly, SA has been reported to improve the emulsifying properties of myofibrillar proteins via electrostatic and hydrophobic interactions [29]. Although the formation of non-covalent QPI/SA complexes has been reported, their application in Pickering emulsions remains underexplored [33]. Notably, Zhong et al. developed a QPI/SA complex-based high-internal-phase emulsion with improved stability and 3D printing performance, highlighting the potential of this biopolymer pair [34]. However, to date, no study has reported the construction of a Pickering emulsion delivery system using QPI/SA complexes. This work represents the first attempt to employ QPI/SA complexes as natural, sustainable, and clean-label stabilizers for curcumin-loaded Pickering emulsions, introducing an alternative to synthetic surfactants and chemical modifications. Unlike previous curcumin-based emulsions, which mostly used animal-derived proteins or single-component stabilizers, our approach leverages the synergistic interaction of quinoa protein and sodium alginate to enhance the interfacial properties, stability, and controlled release. To validate the delivery performance of such emulsions, curcumin (a lipophilic bioactive compound with strong antioxidant, anti-inflammatory, and anticancer properties) was selected as a model compound due to its widespread use in encapsulation research, poor water solubility, environmental instability, and low bioavailability, which make it an ideal candidate for evaluating the controlled release behavior of delivery systems [35,36,37,38]. HeLa cells were chosen for cytotoxicity assessment because they are a well-established model for general cytotoxicity and cancer-related studies, enabling the evaluation of both the safety and potential anticancer activity of the developed emulsions, which aligned with our study objectives.

In this study, QPI/SA complexes were produced through non-covalent interactions. The coacervation behavior of the complexes was investigated across a pH range using turbidimetric titration, and the interaction mechanisms between QPI and SA were further characterized through zeta potential analysis and Fourier-transform infrared (FTIR) spectroscopy. The morphology and surface wettability of the QPI/SA complexes were examined via scanning electron microscopy (SEM) and contact angle measurements. Subsequently, a Pickering emulsion was developed using the QPI/SA complexes as particulate stabilizers, and curcumin, a hydrophobic functional compound, was incorporated as a model bioactive substance. The study focused on exploring the emulsification properties of the QPI/SA complex, assessing the encapsulation of curcumin, and evaluating the emulsion’s structural and physicochemical characteristics following curcumin loading. Additionally, the release behavior of curcumin in simulated gastrointestinal environments and the biocompatibility of the system within a defined polymer concentration range were examined, with further investigation into the potential of the emulsion in anticancer applications.

## 2. Materials and Methods

### 2.1. Materials

Quinoa and corn oil were procured from a local market (Shenyang, China). SA was obtained from McLean Biochemical Technology Co., Ltd. (Shanghai, China). Curcumin (≥98% purity) was supplied by Sinopharm Chemical Reagent Co., Ltd. (Shanghai, China). Fluorescent dyes Nile Blue and Nile Red were purchased from Sima Lab Science Co., Ltd. (Tianjin, China). The Cell Counting Kit-8 (CCK-8) for cytotoxicity assays was acquired from UElandy Biotechnology Co., Ltd. (Suzhou, China). Artificial gastric juice (hydrochloric acid solution, pH = 3.0, pepsin 1% *w*/*v*), artificial intestinal juice (phosphate-buffered solution, pH = 6.8, pancreatin 1% *w*/*v*), and dialysis bags (MWCO: 3.5 kDa) were sourced from Yuanye Biological Co., Ltd. (Shanghai, China). For cell culture experiments, Dulbecco’s Modified Eagle Medium (DMEM), phosphate-buffered saline (PBS), penicillin–streptomycin solution, and 0.25% trypsin–EDTA were purchased from Gibco Life Technologies (Grand Island, NY, USA). Fetal bovine serum (FBS) was obtained from Tianhang Biotechnology Co., Ltd. (Hangzhou, China). All other chemicals and reagents were of analytical grade.

### 2.2. Preparation and Characterization of Quinoa Protein/Sodium Alginate Complexes (QPI/SA)

#### 2.2.1. Preparation of QPI/SA Complexes

The QPI/SA complexes were prepared under different pH conditions according to the earlier method, with slight modifications [34]. Stock solutions (1% *w*/*v*) of QPI and SA were prepared by dissolving appropriate amounts in deionized water under continuous stirring for 3 h, followed by overnight hydration at 4 °C. The QPI and SA solutions were mixed at a 3:1 mass ratio (QPI:SA), followed by pH adjustment using 1 M HCl or 1 M NaOH as required for each target pH (1.6–8.0). The reaction mixture was kept at 20 °C for 1 h to form QPI/SA complexes. The lyophilized complexes were ground into powders and stored in a desiccator until use.

#### 2.2.2. Characterization of QPI/SA Interactions

The intermolecular forces in the QPI/SA complexes were characterized following Feng et al., with modifications [39]. Based on the established turbidity profiles, complex particles formed at selected pH values (1.6, 2.6, 5, 6.7, and 7.5) with a total biopolymer concentration of 0.5% and a QPI/SA mass ratio of 3:1 were analyzed. After pH adjustment, specific denaturants were added to probe different interaction types: 0.1 M NaCl for electrostatic interactions, 0.1 M sodium dodecyl sulfate (SDS) for hydrophobic interactions, and 0.1 M urea for hydrogen bonding. Turbidity measurements were performed for each treatment group using an untreated QPI/SA complex solution as the control.

#### 2.2.3. Turbidity Measurement

The turbidity values were measured using a microplate reader (DR-3000, Wuxi Weidelang Instrument Co., Wuxi, China) at a wavelength of 600 nm.

#### 2.2.4. Zeta Potential Analysis

Zeta potential measurements were conducted using a Nano-ZS90 analyzer (Malvern Instruments Ltd., Malvern, UK) at 25 °C. QPI, SA, and QPI/SA complex solutions were diluted 20-fold with corresponding pH buffers (1.6, 2.6, 5.0, 6.7, 7.5) before analysis. Instrument parameters were set with refractive indices of 1.46 (protein) and 1.33 (aqueous medium).

#### 2.2.5. Fourier-Transform Infrared (FTIR) Spectroscopic Analysis

FTIR spectra were recorded on a Spectrum One spectrometer (PerkinElmer, Shelton, CT, USA) using the KBr pellet method. Briefly, 1 mg of sample was thoroughly mixed with 99 mg of spectroscopic-grade KBr and pressed into transparent pellets. Spectra were acquired in the range of 400–4000 cm^−1^ with 16 scans at a resolution of 4 cm^−1^, using air as a background reference.

#### 2.2.6. Scanning Electron Microscopy (SEM)

Morphological characterization was performed using an S-4800 SEM instrument (Hitachi, Hitachi, Japan) at 1.00 kV acceleration voltage. Samples were sputter-coated with gold prior to imaging at 250× and 15,000× magnifications.

#### 2.2.7. Three-Phase Contact Angle Measurement

Contact angle measurements were conducted following Bai et al. using a DSA100 instrument (KRUSS, Hagen, Germany) [40]. Freeze-dried samples were compressed into discs with a 13 mm diameter × 1 mm thickness. Deionized water droplets were deposited on sample surfaces and imaged at 10 fps.

### 2.3. Preparation and Stability of Pickering Emulsions

#### 2.3.1. Emulsion Preparation

Pickering emulsions were prepared by homogenizing corn oil (oil phase) with the aqueous phase (1% *w/v* QPI/SA complex solutions) at 13,000 rpm for 3 min using a high-speed homogenizer (IKA T25, Staufen, Germany) [36]. The oil-to-water ratio was varied to optimize the emulsion stability.

#### 2.3.2. Creaming Stability Assessment

The creaming stability of the Pickering emulsions was evaluated by storing samples in graduated test tubes at 20 °C. The creaming index, defined as the ratio of the emulsion layer height (H_0_) to the total emulsion height (H), was calculated as follows:

(1) $$E\mathrm{mulsion}\;\mathrm{index}\;(\%)\;=\;\frac{H_{0}}{H}\times \;100$$

#### 2.3.3. Emulsion Droplet Size Analysis

The emulsion droplet size distribution was characterized by laser diffraction (Mastersizer 3000, Malvern Instruments, UK), using refractive indices of 1.46 (dispersed phase) and 1.33 (continuous phase). Volume-weighted (D_4,3_) and surface-weighted (D_3,2_) mean diameters were recorded at 7% obscuration.

### 2.4. Preparation and Characterization of Curcumin-Loaded Pickering Emulsions

#### 2.4.1. Curcumin-Loaded Pickering Emulsions

Curcumin-loaded Pickering emulsions were prepared by homogenizing (13,000 rpm, 3 min) 30% and 60% (*v*/*v*) curcumin-enriched corn oil (4 mg/mL, clarified by centrifugation at 8000× *g* for 20 min) with QPI/SA complex solutions (5:1 mass ratio, pH 5, 1% *w*/*v*) [36].

#### 2.4.2. In Vitro Release of Curcumin

To simulate gastric digestion, 5 mL of the curcumin-loaded emulsion was mixed with 5 mL of artificial gastric fluid and sealed in a dialysis bag (MWCO 3500 Da). The bag was immersed in 1 L of buffer solution (maintaining an identical pH and ionic composition to the artificial gastric fluid) and incubated at 37 °C with shaking at 100 rpm for 2 h. For intestinal digestion, the pH of the mixture was adjusted to 7.0, followed by the addition of 10 mL artificial intestinal fluid. The dialysis bag was again incubated in 1 L buffer solution (maintaining an identical pH and ionic composition to the artificial intestinal fluid) at 37 °C and 100 rpm for an additional 2 h [41].

At specified time intervals (0, 30, 60, 90, and 120 min), 100 μL of the digestion medium was sampled, mixed with 900 μL anhydrous ethanol, and centrifuged at 9000 rpm for 5 min, and the supernatant was diluted tenfold. The absorbance was measured at 425 nm, and the curcumin concentration was calculated using a standard curve. The release rate of curcumin was calculated using the following formula:

(2) $$\mathrm{Curcumin}\;\mathrm{release}\;\mathrm{rate}\;(\%)\;=\;\left(1-\frac{V}{V_{0}}\right)\times 100$$

where V is the curcumin content at a given digestion time, and V_0_ is the initial curcumin content in the emulsion.

#### 2.4.3. Confocal Laser Scanning Microscopy Analysis

The spatial distribution of emulsion components was analyzed by confocal laser scanning microscopy (CLSM). Prior to emulsion preparation, the oil phase was labeled with Nile Red (0.1% *w*/*v*) and the aqueous phase with Nile Blue (0.1% *w*/*v*). Samples were imaged using excitation wavelengths of 514 nm (Nile Red) and 633 nm (Nile Blue) to differentiate phases.

#### 2.4.4. In Vitro Cytotoxicity

The cytotoxicity of the QPI, QPI/SA complexes, and curcumin-loaded Pickering emulsions was evaluated using the CCK-8 assay, with modifications based on Zhang et al. [38]. HeLa cells were cultured in DMEM supplemented with 10% FBS and 1% penicillin–streptomycin under standard conditions (37 °C, 5% CO_2_). Cells in the logarithmic growth phase were digested with 0.25% trypsin–EDTA to obtain a single-cell suspension, which was seeded into 96-well plates at a density of 5 × 10^3^ cells/well. Sterile PBS (pH 7.4) was added to the outer wells to reduce evaporation. After 24 h of incubation, cells were treated with various concentrations of QPI and QPI/SA solutions (diluted in culture medium) to assess cytotoxicity. The antitumor activity of the curcumin-loaded Pickering emulsions was evaluated under the same conditions.

After 12 h of treatment, the medium was replaced with fresh medium containing 10% CCK-8 reagent. Cells were incubated at 37 °C in the dark for 2 h, and the absorbance was measured at 450 nm using a microplate reader. Cell viability was calculated using the following formula:

(3) $$\mathrm{Cell}\;\mathrm{viability}\;\left(\%\right)\;=\;\frac{A_{1}-A_{0}}{A_{2}-A_{0}}\times 100$$

where A_1_ is the absorbance of the treated cells, A_2_ is the absorbance of the untreated control, and A_0_ is the absorbance of the blank (medium only).

### 2.5. Statistical Analysis

The experiments were performed in triplicate, and the results were analyzed using one-way analysis of variance and Duncan’s multiple range test in SPSS 26.0. Statistical significance was set at *p* < 0.05. Results are presented as the mean ± standard deviation.

## 3. Results and Discussion

### 3.1. Characterization of Quinoa Protein/Sodium Alginate Complexes (QPI/SA)

#### 3.1.1. Molecular Interactions in QPI/SA Complexes

**Figure 1:**
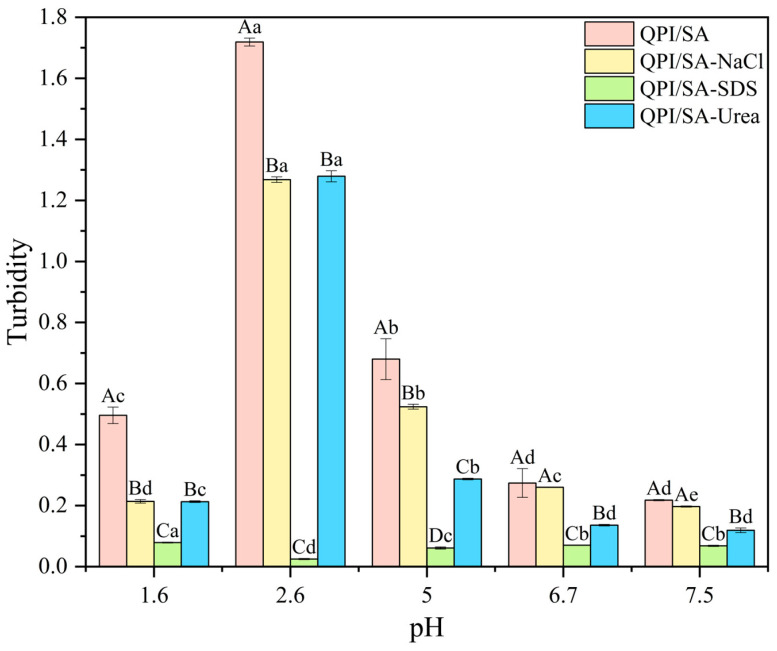
Effects of different denaturants on the turbidity of QPI/SA complexes. Lowercase letters (a–d) indicate significant differences in the turbidity of the same QPI/SA complex across different pH values, while uppercase letters (A–D) indicate significant differences among QPI/SA complexes treated with different denaturants at the same pH.

**Figure 2:**
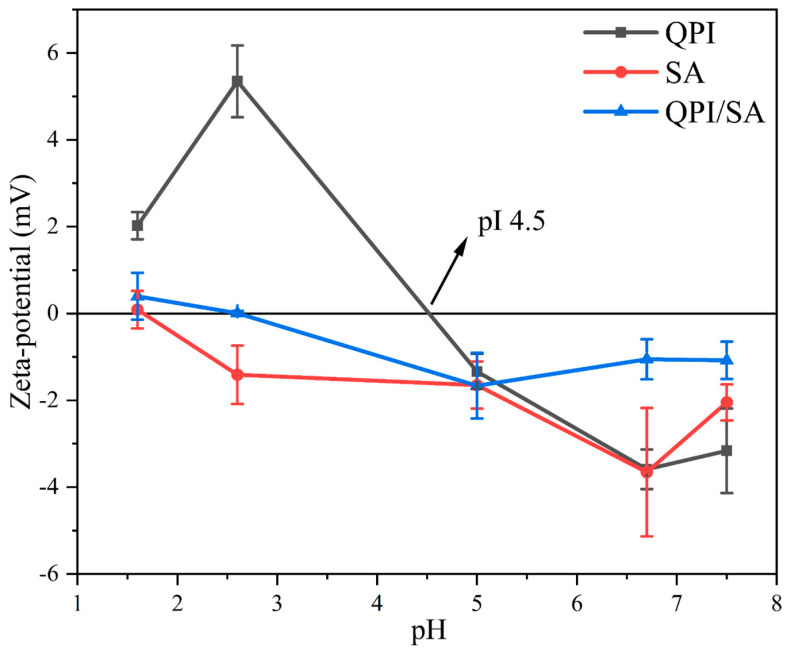
pH-dependent zeta potential profiles of QPI, SA, and QPI/SA complexes.

To investigate the non-covalent interactions involved in the formation of QPI/SA complexes, the effects of different denaturants on solution turbidity were evaluated at various pH levels (Figure 1). Specifically, 0.1% NaCl was used to modulate electrostatic interactions, 0.1% SDS to disrupt hydrophobic interactions, and 0.1% urea to interfere with hydrogen bonding [42]. Compared to the untreated control, the three denaturants significantly reduced the turbidity of the QPI/SA complex solutions at pH 1.6, 2.6, and 5.0, respectively (*p* < 0.05), indicating the coexistence of electrostatic, hydrophobic, and hydrogen-bonding interactions in complex formation under these conditions. At pH 6.7 and 7.5, NaCl had no significant effect on turbidity, suggesting weaker electrostatic interactions, while the significant turbidity reductions following SDS and urea treatment indicated that hydrophobic interactions and hydrogen bonding were the dominant forces stabilizing the complexes at a near-neutral pH [43]. Turbidity was minimal at pH values close to neutral (6.7, 7.5), as both QPI and SA had a significant negative charge, resulting in strong electrostatic repulsion between them, which inhibited the formation of QPI/SA complexes. As the pH decreased, the turbidity of the QPI/SA solution increased. At pH 5, although both QPI and SA had net negative charges (Figure 2), the electrostatic attraction between the anionic groups on the SA and cationic patches on the surface of QPI could form electrostatic complexes [44]. The turbidity reached its maximum at pH 2.6 because, at this pH, QPI and SA had opposite charges, resulting in electrostatic attraction between them, which promoted the formation of QPI/SA complexes. At pH 6, SA was protonated, approaching a neutral charge. This reduced the electrostatic attraction between SA and QPI, preventing the formation of QPI/SA complexes and decreasing the turbidity. Liu et al. reported similar findings when investigating the effect of pH on the solubility of whey protein isolate/sodium alginate complexes [45]. Based on these results, it can be inferred that the solubility of QPI/SA complexes is pH-dependent, a characteristic that suggests their potential as functional carriers for targeted bioactive compound release in specific pH environments, such as the intestines.

#### 3.1.2. Zeta Potential

Zeta potential analysis was conducted to evaluate the surface charge distribution and interaction forces of particles in solution [46]. As shown in Figure 2, the absolute zeta potential of QPI first decreased and then increased as the pH decreased. At the isoelectric point (pI ≈ 4.5) of QPI, the zeta potential approached 0 mV, indicating minimal surface charge and a high tendency for protein aggregation due to reduced electrostatic repulsion [47]. In contrast, SA generally exhibited negative charges across the tested pH range, although its zeta potential approached neutrality at strongly acidic conditions (pH 1.6), which can be attributed to the protonation of carboxyl groups under a low pH. Its absolute zeta potential generally increased with a rising pH, but, at pH 7.5, a statistically significant reduction (*p* < 0.05) was observed, possibly due to intramolecular hydrogen bonding between –COO^−^ and –OH groups, which may have affected charge distribution [48]. For the QPI/SA complexes, the absolute zeta potential consistently fell between those of QPI and SA, suggesting electrostatic interactions and partial charge neutralization. As the pH increased, the zeta potential of the complex first decreased, reaching zero at a pH of approximately 2.6, where the electrostatic attraction between oppositely charged groups was maximal, promoting particle aggregation. At pH 5.0, the complex exhibited the highest absolute negative potential, suggesting enhanced colloidal stability due to increased electrostatic repulsion. When the pH exceeded 5.0, the absolute potential decreased again, indicating weakened protein–polysaccharide interactions and the transition of the system into a co-soluble state [34].

#### 3.1.3. Fourier-Transform Infrared

**Figure 3:**
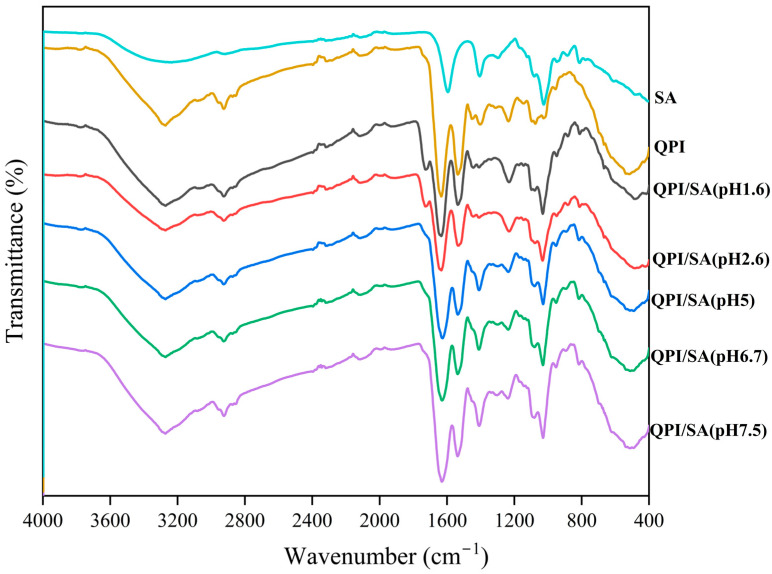
pH-dependent Fourier-transform infrared spectral characteristics of QPI, SA, and QPI/SA complexes.

As illustrated in Figure 3, the FTIR spectra of the QPI, SA, and QPI/SA complexes at various pH values provide insight into their intermolecular interactions. In the QPI spectrum, the broad absorption band observed at 3600–3100 cm^−1^ corresponds to the stretching vibrations of O–H, C–H, and hydrogen bonds within or between protein molecules. The amide I band (1700–1600 cm^−1^) represents C=O stretching vibration, the amide II band (1575–1480 cm^−1^) is attributed to N–H bending, and the amide III band (1400–1200 cm^−1^) is associated with C–N stretching and N–H bending vibrations, which are characteristic of protein secondary structures [46,49]. For SA, typical peaks include strong O–H stretching vibration at 3233 cm^−1^, a –CH_2_ stretching peak at 2918 cm^−1^, symmetric and asymmetric stretching vibrations of –COO^−^ at 1640–1600 cm^−1^ and 1460–1400 cm^−1^, respectively, and a C–O–C stretching peak in the 1100–1000 cm^−1^ region, confirming the presence of polysaccharide backbone structures [50,51]. Compared with the individual components, the QPI/SA complex exhibited more intense O–H stretching vibration in the 3600–3100 cm^−1^ range, suggesting the formation of hydrogen bonds between QPI and SA. Additionally, the amide I band of the complex showed a redshift with the increasing pH, indicating hydrogen bond formation between the C=O groups of QPI and the O–H groups of SA. The amide II band showed decreased intensity and a blueshift, reflecting alterations in the amino groups of QPI and further suggesting hydrogen bonding between amino and carboxyl groups. Furthermore, the appearance of a characteristic absorption peak near 1025 cm^−1^, attributed to the C–O–C bond of SA, provides additional evidence for complex formation [52]. Interestingly, at pH 2.6, the O–H stretching band shifted toward a higher wavenumber, indicating strong electrostatic interactions between QPI and SA, whereas, at other pH values, redshifts were observed, likely due to changes in the hydrogen-bonding environment and weaker electrostatic interactions [51]. Moreover, a weak absorption peak at approximately 1726 cm^−1^ appeared at pH 1.6 and 2.6, which can be attributed to the protonation of –COO^−^ groups forming –COOH. This peak disappeared at pH ≥ 5 and was replaced by a band near 1400 cm^−1^ associated with deprotonated carboxylate groups (COO^−^), further confirming the pH-dependent structural changes in the QPI/SA complexes. Collectively, these results demonstrate that hydrogen bonding and electrostatic interactions are critical to the formation and stability of QPI/SA complexes across varying pH levels.

#### 3.1.4. SEM

**Figure 4:**
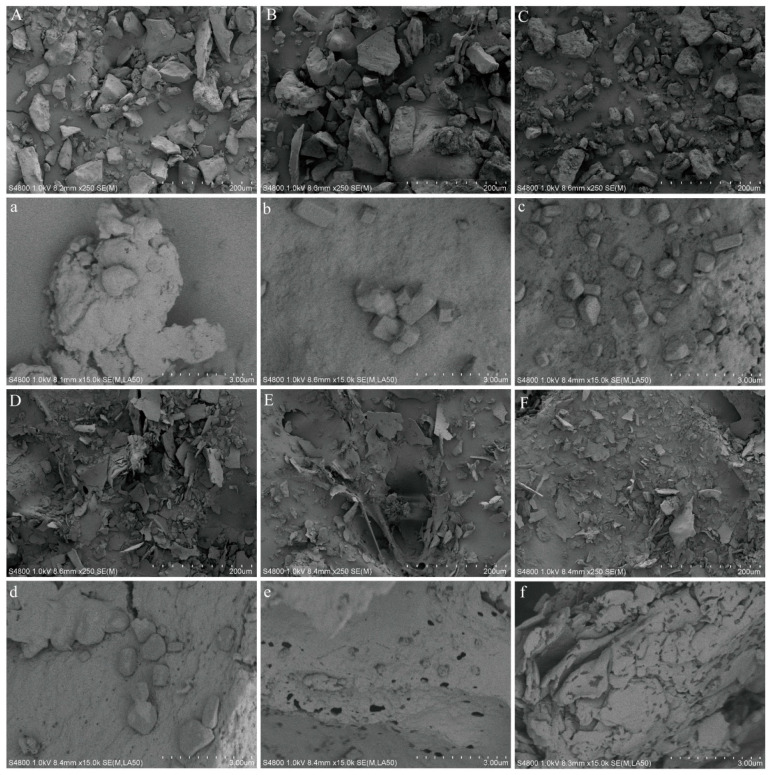
Scanning electron micrographs of QPI and QPI/SA complexes under varying pH conditions. (**A**,**a**) QPI (pH 5.0); (**B**,**b**) QPI/SA (pH 1.6); (**C**,**c**) QPI/SA (pH 2.6); (**D**,**d**) QPI/SA (pH 5.0); (**E**,**e**) QPI/SA (pH 6.7); (**F**,**f**) QPI/SA (pH 7.5).

The microstructural morphologies of QPI/SA complexes prepared under different pH conditions were examined using scanning electron microscopy (SEM), as shown in Figure 4. At pH 5.0, QPI exhibited large, irregularly shaped aggregates with rough surfaces, which can be attributed to the proximity of this pH to the isoelectric point of the protein, leading to reduced electrostatic repulsion and enhanced protein–protein aggregation. Upon complexation with SA, the morphologies of the QPI/SA complexes varied significantly with the pH. At pH 1.6, the polysaccharide was highly protonated, weakening the electrostatic interaction with the protein and resulting in the partial dissociation of the complexes. Consequently, the complexes exhibited a more compact structure with minimal porosity compared to QPI alone. As the pH increased, the interaction between QPI and SA was enhanced due to increased charge differences, leading to the formation of more granular complexes with progressively denser porous structures. Notably, at pH 7.5, the QPI/SA complex entered the soluble complex phase, and large aggregates were no longer observed. Instead, the complexes exhibited continuous sheet-like structures with smooth surfaces, suggesting weaker intermolecular interactions and a more dispersed system under near-neutral conditions. These observations highlight the pH-dependent assembly behavior of QPI/SA complexes and the critical role of electrostatic interactions in determining their microstructural characteristics.

#### 3.1.5. Three-Phase Contact Angle

**Figure 5:**
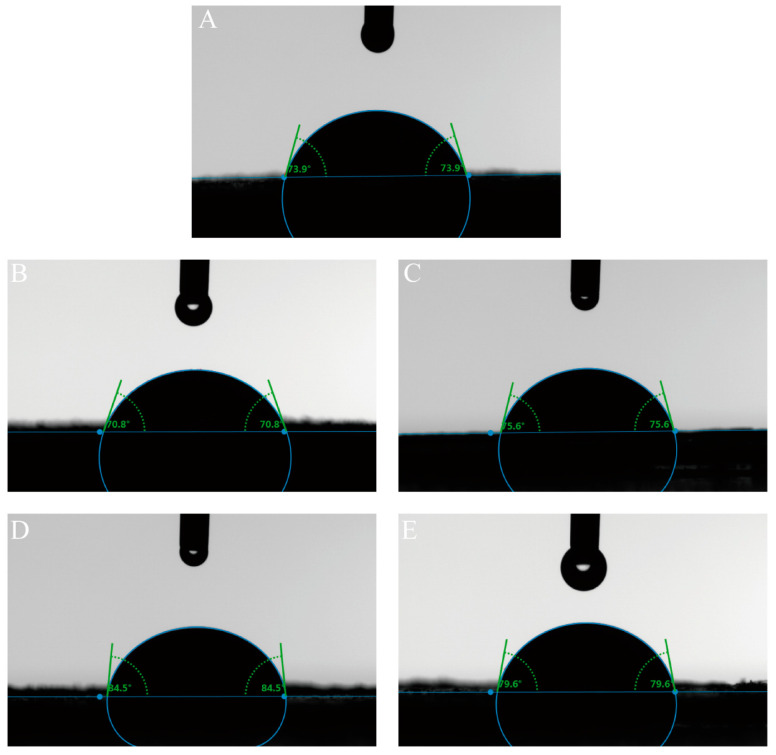
pH-dependent three-phase contact angle measurements of QPI and QPI/SA complexes. (**A**) QPI; (**B**) QPI/SA (pH 2.6); (**C**) QPI/SA (pH 5.0); (**D**) QPI/SA (pH 6.7); (**E**) QPI/SA (pH 7.5).

The three-phase contact angle is a key indicator of the wettability of solid particles in immiscible systems and directly influences their ability to adsorb at the oil–water interface, which in turn influences the stability of Pickering emulsions [53,54]. As shown in Figure 5, both QPI and QPI/SA composite particles exhibited contact angles below 90°, confirming their hydrophilic nature. Pure QPI particles displayed a contact angle of 73.9°, while the introduction of hydroxyl and carboxyl groups from SA modified the wettability of the composite particles. Specifically, the QPI/SA composites formed at pH 2.6, 5.0, 6.7, and 7.5 exhibited contact angles of 70.8°, 75.6°, 84.5°, and 79.6°, respectively. These results indicate that QPI/SA composites’ hydrophobicity increases under near-neutral and alkaline conditions. Notably, the contact angle at pH 6.7 was closest to 90°, indicating an optimal amphiphilic balance and the strongest potential for interfacial adsorption, favoring stable Pickering emulsion formation.

### 3.2. Effects of Different Conditions on Pickering Emulsion Properties

#### 3.2.1. Effect of QPI/SA Mass Ratio on Physicochemical Properties of Pickering Emulsions

**Figure 6:**
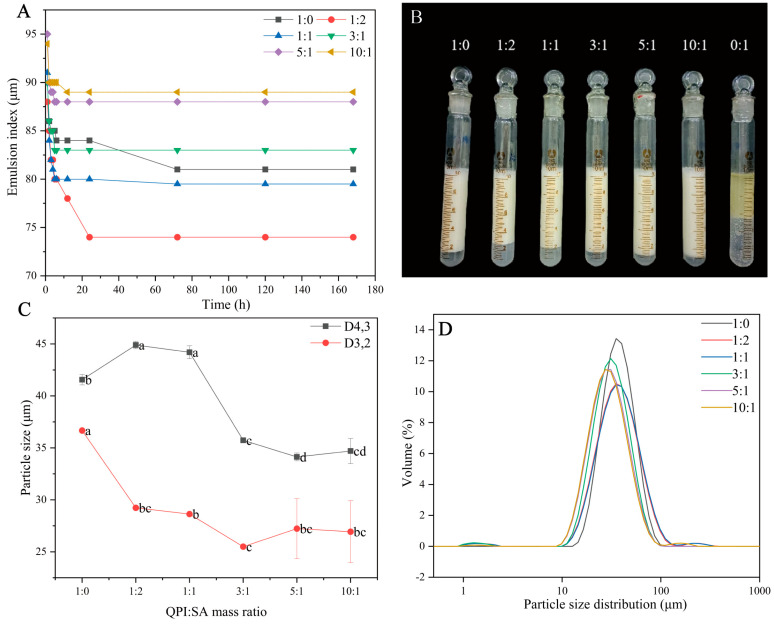
QPI/SA mass ratio effects on the (**A**) emulsion stability index, (**B**) visual stability after 24 h, (**C**) emulsion droplet size, and (**D**) particle size distribution (D_4,3_). Where, different superscript letters indicate significant differences (*p* < 0.05).

The emulsification index is a direct and reliable parameter for evaluating emulsion stability, with higher values generally indicating better stability. In this study, the effects of different mass ratios of QPI to SA on the stability of the Pickering emulsions were investigated under fixed conditions: a 0.5% total biopolymer concentration, a 50% oil-phase volume, and pH 5. QPI and SA alone, each at the same total concentration, served as controls. As shown in Figure 6A,B, SA alone was unable to stabilize the emulsions, while QPI alone formed an emulsion layer with moderate stability, consistent with previous findings [55]. The emulsification indices of the QPI/SA complexes varied depending on their mass ratios. With increasing QPI proportions, both the emulsification index and stability of the resulting emulsions significantly improved. At QPI/SA mass ratios of 1:1 and 1:2, the emulsification indices were lower than that of QPI alone. However, when the ratio increased to 3:1, the emulsion exhibited better stability than the QPI-based emulsion, and, at 10:1, the emulsion demonstrated the highest emulsification index and excellent long-term stability, maintaining a value of 89% after 7 days of storage. These results suggest that higher QPI content in the complex enhances the emulsion stability. Furthermore, the droplet size of the emulsions was analyzed using a laser particle size analyzer. As shown in Figure 6C,D, all emulsions exhibited micron-scale droplet sizes, and the average droplet size (D_3,2_ and D_4,3_) decreased with increasing QPI content in the complex. At a QPI/SA ratio of 1:2, the emulsion had a D_4_,_3_ of 44.20 ± 0.62 μm, which gradually decreased to 35.73 ± 0.11 μm, 34.13 ± 0.38 μm, and 34.70 ± 1.21 μm at mass ratios of 3:1, 5:1, and 10:1, respectively—values that were all smaller than that of the QPI-only emulsion. The span indicates the droplet size distribution; the lower the span value, the more homogenous the emulsion. The QPI emulsion exhibited the lowest span value (0.98). The span values of the QPI/SA emulsions, while all greater than that of the QPI emulsion, remained below 1.3, suggesting relatively narrow droplet size distributions. Among them, the QPI/SA (3:1) emulsion had the smallest span value of 1.094. Notably, the droplet size and span value slightly increased at the highest QPI ratio (10:1), potentially due to particle aggregation or insufficient interfacial coverage. The particle size distribution remained unimodal, and an increasing pH shifted the distribution toward smaller sizes, indicating that an appropriate protein-to-polysaccharide ratio is beneficial for stabilizing emulsions. These findings align with those of Kim et al., who reported enhanced emulsion stability through the incorporation of sodium alginate into whey/pea protein systems, facilitating protein adsorption at the oil–water interface [56].

#### 3.2.2. pH-Dependent Behavior of QPI/SA Complex-Stabilized Pickering Emulsions

**Figure 7:**
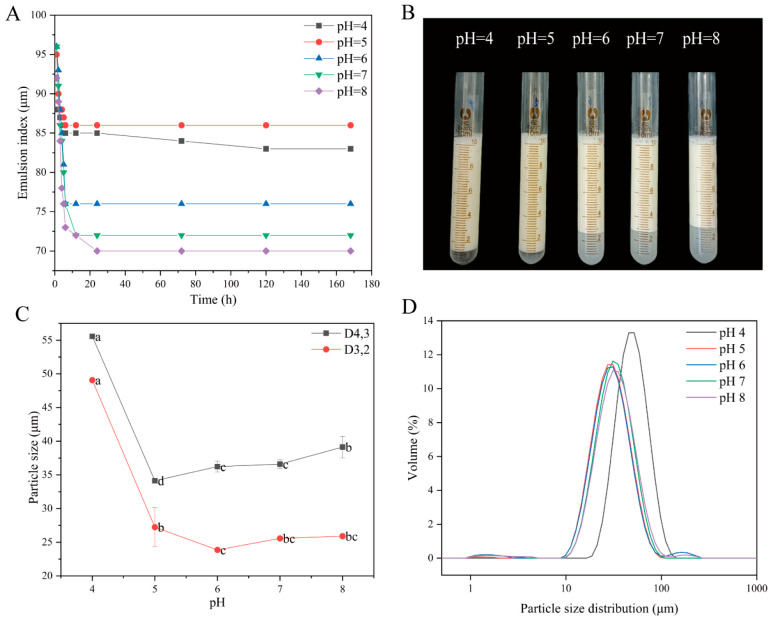
pH-dependent characteristics of QPI/SA-stabilized Pickering emulsions: (**A**) emulsion stability index, (**B**) visual stability after 24 h, (**C**) emulsion droplet size, and (**D**) particle size distribution (D_4,3_). Where, different superscript letters indicate significant differences (*p* *<* 0.05).

The Pickering emulsions stabilized by QPI/SA complexes prepared at different pH values were uniformly designated as QPI/SA _(pH)_ PEs. As shown in Figure 7, the emulsification stability of QPI/SA (5:1) complexes, prepared under various pH conditions with a total biopolymer concentration of 0.5% (*w*/*w*) and an oil-phase volume of 50%, was systematically investigated. QPI/SA complexes formed in the pH range of 4–8 were able to stabilize emulsions to varying extents. As depicted in Figure 7A,B, the emulsification index of the Pickering emulsions initially increased and then decreased with the rising pH. Over the first 24 h, the emulsification index gradually declined before stabilizing. Notably, the QPI/SA _(4)_ PEs and QPI/SA _(5)_ PEs exhibited superior emulsification indices under weakly acidic conditions, with the QPI/SA _(5)_ PEs maintaining the highest emulsification index of 86% after 7 d of storage, while that of the QPI/SA _(4)_ PEs decreased slightly to 83%, indicating excellent long-term stability. Droplet size analysis (Figure 7C,D) further revealed that the particle sizes of the emulsions followed a similar trend to that of the emulsification index, decreasing initially with an increasing pH and then increasing again. The QPI/SA _(4)_ PEs exhibited the largest droplet size (D_4,3_ = 55.56 ± 0.12 μm), while the QPI/SA _(5)_ PEs had the smallest droplet size (D_4,3_ = 34.13 ± 0.38 μm). The span values for the QPI/SA emulsions were as follows: QPI/SA _(4)_ PEs = 0.979, QPI/SA _(5)_ PEs = 1.161, QPI/SA _(6)_ PEs = 1.215, QPI/SA _(7)_ PEs = 1.707, and QPI/SA _(8)_ PEs = 1.225. The QPI/SA _(4)_ PEs and QPI/SA _(5)_ PEs exhibited narrower droplet size distributions. While the QPI/SA _(4)_ PEs had the lowest span value, their larger average droplet size (Figure 7C) resulted in poorer emulsion stability compared to QPI/SA (5) (Figure 7A,B). This enhanced stability at pH 5.0 was attributed to the increased net surface charge on the QPI/SA complexes, which increased the electrostatic repulsion at the oil–water interface, thus effectively preventing droplet coalescence [57]. These results indicate that the pH plays a critical role in modulating the interfacial behavior of QPI/SA complexes, and pH 5 is optimal for achieving a minimal droplet size and maximum emulsion stability.

#### 3.2.3. Influence of Oil-Phase Volume Fraction on Pickering Emulsion Stability

**Figure 8:**
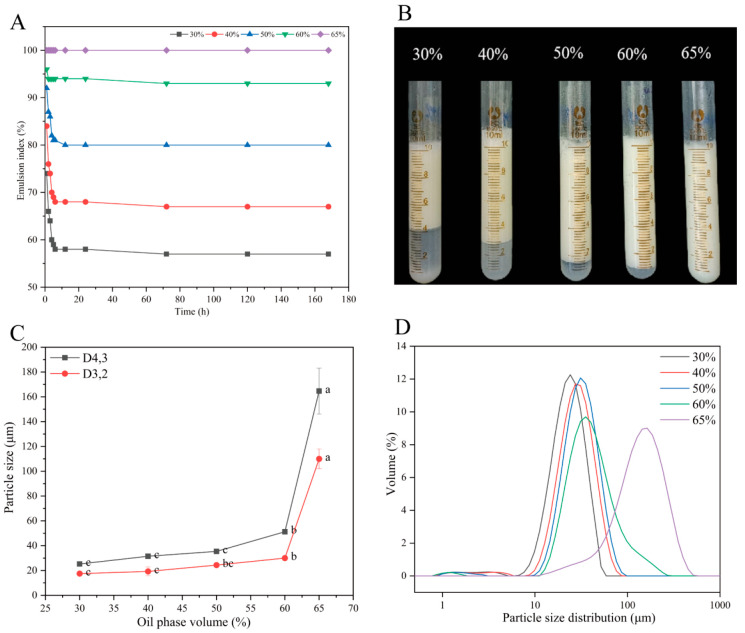
Oil-phase volume-dependent characteristics of QPI/SA-stabilized Pickering emulsions: (**A**) emulsion stability index, (**B**) visual stability after 24 h, (**C**) emulsion droplet size, and (**D**) particle size distribution (D_4,3_). Where, different superscript letters indicate significant differences (*p* *<* 0.05).

Figure 8 illustrates the influence of different oil-phase volumes on the properties of Pickering emulsions (PEs), with preparation conditions of a 0.5% (*w*/*w*) total biopolymer concentration, pH 5.0, and a QPI/SA mass ratio of 5:1. The emulsions were denoted as QPI/SA _(φ)_ PEs, where φ represents the oil-phase volume. As shown in Figure 8A,B, increasing the oil-phase volume from 30% to 65% led to a corresponding increase in the initial emulsion layer volume and emulsification index, indicating an enhanced emulsification capacity. Over time, all emulsions except the QPI/SA _(φ = 65%)_ PEs exhibited varying degrees of phase separation; however, stabilization was observed after 24 h, with minimal further changes in the emulsion layer volume. Notably, the QPI/SA _(φ = 60%)_ PEs exhibited slight aqueous phase separation after 24 h but maintained a high emulsification index of 93% after 7 days, suggesting good long-term stability. Interestingly, the QPI/SA _(φ = 65%)_ PEs remained visually homogeneous without noticeable stratification, implying the potential transition of the emulsion system into a semi-solid gel state under high internal phase conditions [58]. As presented in Figure 8C,D, the average droplet size of the emulsions (D_4_,_3_) increased progressively with the rise in oil-phase volume. For instance, the QPI/SA _(φ = 30%)_ PEs showed the smallest particle size (25.3 ± 0.02 μm), while increasing the oil-phase volume from 40% to 65% led to a gradual increase in droplet size. At φ = 65%, the emulsion exhibited a substantial increase in droplet size (164.67 ± 18.56 μm). This phenomenon can be attributed to the fixed amount of QPI/SA complex available in the system: at lower oil-phase volumes, more particles can adsorb per unit oil–water interfacial area, resulting in higher surface coverage and smaller droplets. As the oil-phase volume increases, the interfacial area expands, reducing the surface coverage of solid particles and leading to larger droplets under high shear conditions [59]. The significant droplet size increase observed in the QPI/SA _(φ = 65%)_ PEs may also be associated with the transition of the emulsion into a semi-solid gel network, which limits droplet mobility and stabilizes the system. The QPI/SA _(φ = 30%)_, QPI/SA _(φ = 40%)_, and QPI/SA _(φ = 50%)_ emulsions had similar span values of 1.062, 1.121, and 1.094, respectively. The QPI/SA _(φ = 60%)_ and QPI/SA _(φ = 65%)_ emulsions had larger span values of 1.755 and 1.509, respectively, indicating that their droplet size distributions were wider, which is consistent with the results shown in Figure 8D. These results suggest that the emulsion stability depends not only on the average droplet size but also on the droplet size distribution.

### 3.3. Characterization of Curcumin-Loaded Pickering Emulsions

#### 3.3.1. In Vitro Release Kinetics Analysis

**Figure 9:**
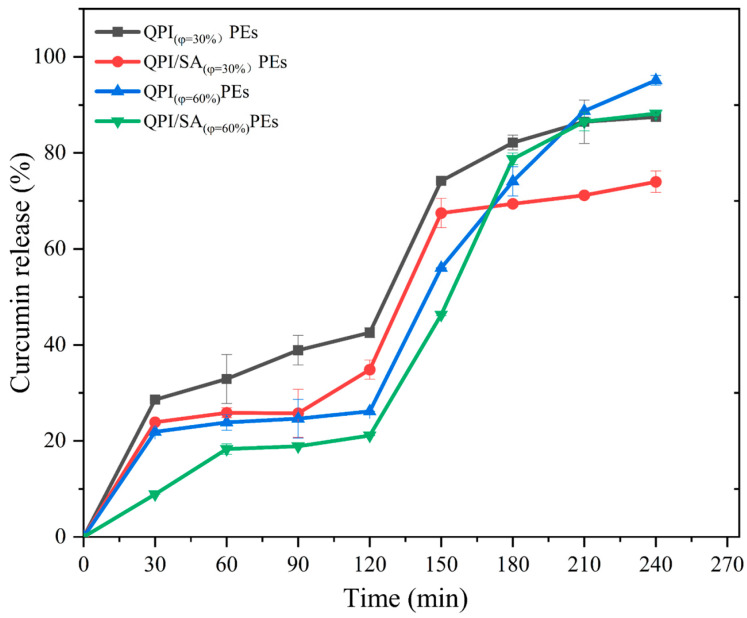
Rates of curcumin release in Pickering emulsions containing 30% and 60% oil-phase volumes prepared by QPI/SA at different stages of simulated digestion.

As shown in Figure 9, curcumin encapsulated in the four different Pickering emulsions exhibited distinct release profiles during simulated gastrointestinal digestion. After gastric digestion, the curcumin release rates for the QPI _(φ = 30%)_ PEs, QPI/SA _(φ = 30%)_ PEs, QPI _(φ = 60%)_ PEs, and QPI/SA _(φ = 60%)_ PEs were 42.58 ± 1.02%, 34.87 ± 2.01%, 26.17 ± 0.02%, and 21.13 ± 0.02%, respectively. Under the same oil-phase volume, emulsions stabilized by QPI/SA complexes exhibited lower curcumin release than those stabilized by QPI alone, likely due to the enhanced structural integrity and resistance of QPI/SA complexes to enzymatic hydrolysis, which aligns with previous reports on protein–polysaccharide complexes exhibiting stronger gastric stability compared to single-protein systems [60]. Furthermore, emulsions prepared with lower oil-phase volumes (φ = 30%) released curcumin more rapidly during gastric digestion due to their smaller droplet sizes and larger specific surface areas, which facilitated greater exposure to digestive enzymes. In contrast, during intestinal digestion, the release rate of curcumin was significantly enhanced in emulsions with a higher oil-phase volume. Specifically, QPI _(φ = 60%)_ PEs-cur showed the highest cumulative release (95.12 ± 1.00%), followed by QPI/SA _(φ = 60%)_ PEs-cur (88.21 ± 0.01%), QPI _(φ = 30%)_ PEs-cur, and QPI/SA _(φ = 30%)_ PEs-cur (73.99 ± 2.25%). Similar trends have been observed in whey protein–alginate Pickering emulsions, where higher lipid content accelerated lipolysis and promoted bioactive release during intestinal digestion [61]. This increase in the release rate with higher oil content is likely due to the hydrolysis of lipid droplets by pancreatic lipase, which disrupts the emulsion structure and facilitates curcumin diffusion [62]. These findings demonstrate that the QPI/SA complexes enhance both the emulsion stability and controlled curcumin release, with the release profile being further influenced by the oil-phase volume of the emulsion system.

#### 3.3.2. Microstructural Characterization by Confocal Laser Scanning Microscopy (CLSM)

**Figure 10:**
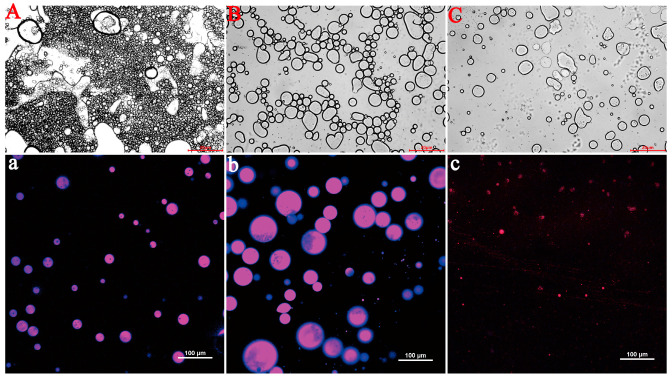
Structural evolution of 60% oil-phase Pickering emulsions during simulated GI digestion: (**A**,**a**) initial state; (**B**,**b**) post-gastric phase (SGF, 2 h); (**C**,**c**) post-intestinal phase (SIF, 2 h).

The microstructure of QPI/SA _(φ = 60%)_ PEs-cur after gastric and intestinal digestion was further observed by laser confocal microscopy and optical microscopy (Figure 10). After gastric digestion, the droplet size increased significantly, and the emulsion stability decreased. This could be attributed to the flocculation and aggregation of droplets caused by the low pH of the simulated gastric fluid (SGF), which altered the surface structure of the emulsifier particles [38]. Additionally, the degradation of QPI/SA complexes by pepsin may have disrupted the integrity of the composite particles, further compromising the emulsion stability [63]. Following simulated intestinal fluid (SIF) digestion, significant droplet coalescence and a reduction in droplet number were observed, likely due to the enzymatic hydrolysis of the QPI/SA complex and oil phase by trypsin and pancreatic lipase [63,64]. Similar destabilization phenomena have been observed in chitosan/gum arabic- and cellulose-based Pickering emulsions loaded with curcumin, where enzymatic degradation and bile salts reduced droplet stability during intestinal digestion [65]. These structural changes during digestion confirm the susceptibility of QPI/SA-stabilized emulsions to physiological conditions and highlight the dynamic nature of their protective and release behaviors.

#### 3.3.3. Evaluation of Anticancer Efficacy

**Figure 11:**
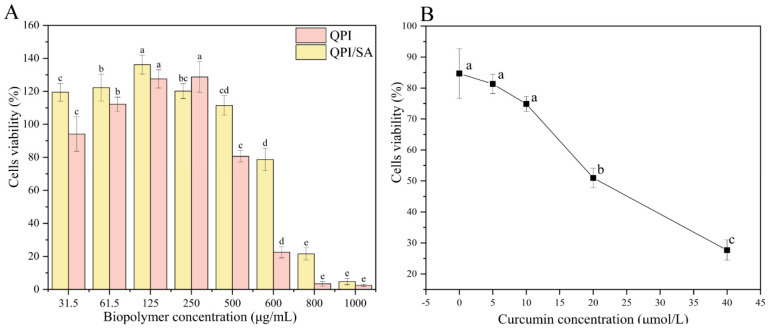
In vitro anticancer activity: (**A**) viability impact of protein–polysaccharide complexes; (**B**) therapeutic potential of curcumin-loaded emulsions (60% oil phase) at varying concentrations. Where, different superscript letters indicate significant differences (*p* *<* 0.05).

Cytotoxicity testing is essential for evaluating the potential applications of curcumin-loaded Pickering emulsions in health foods and biomedical fields. As shown in Figure 11A, both QPI and QPI/SA exhibited a growth-promoting effect on HeLa cells when the biopolymer concentration was below 500 μg/mL, possibly due to the supply of nutrients such as amino acids and trace elements by QPI or QPI/SA at low concentrations, thereby promoting cellular proliferation [66]. However, at concentrations exceeding 500 μg/mL, both biopolymers demonstrated significant cytotoxicity in a dose-dependent manner. This inhibitory effect may be due to high protein concentrations disrupting cellular homeostasis, thereby impairing cellular function or exerting toxic effects. These findings suggest that QPI and QPI/SA complexes exhibit good biocompatibility at concentrations ≤500 μg/mL, but their cytotoxicity becomes evident at higher concentrations.

Based on these results, Pickering emulsions loaded with different concentrations of curcumin (QPI/SA (φ = 60%) PEs-cur) were prepared using a QPI/SA complex solution at a safe biopolymer concentration of 125 μg/mL to investigate their cytotoxicity against HeLa cells (Figure 11B). QPI/SA PEs without curcumin exhibited low toxicity, maintaining cell viability of 84.68%, well above the 70% viability threshold for cytocompatibility. However, with increasing curcumin concentrations in the emulsion, a gradual decrease in HeLa cell viability was observed. When the curcumin concentration reached 20 μmol/L, cell viability declined to 50.96%, indicating a significant inhibitory effect. This dose-dependent cytotoxicity is consistent with observations in other curcumin-based Pickering systems, such as zein/pectin or chitosan/gum arabic emulsions, where the anticancer activity increased with curcumin loading [65,67]. These results indicate that QPI/SA-stabilized Pickering emulsions are mildly cytotoxic, and their anticancer activity is enhanced with increasing curcumin loading, highlighting their potential as functional carriers in food and biomedical applications.

## 4. Conclusions

A novel quinoa protein isolate/sodium alginate (QPI/SA) composite particle system was developed and applied as a Pickering stabilizer for bioactive compound delivery. The QPI/SA complex was formed through non-covalent interactions between protein and polysaccharide components, and its coacervation behavior was characterized through turbidimetric titration. Comprehensive structural analyses, including zeta potential, FTIR spectroscopy, and three-phase contact angle measurements, revealed that the formation of QPI/SA complexes involved electrostatic interactions, hydrophobic interactions, and hydrogen bonding under acidic conditions, while hydrophobic and hydrogen bonding predominated under neutral and alkaline conditions. Scanning electron microscopy and three-phase contact angle measurements further confirmed that complexation led to changes in surface morphology and improved hydrophilicity. Pickering emulsions were subsequently prepared using QPI/SA composites as emulsifiers. The emulsification performance analysis showed that emulsions formed at pH 5 with a QPI/SA mass ratio of 5:1 and an oil-phase volume fraction of 60% exhibited optimal stability, maintaining an emulsification index exceeding 90% after 7 days of storage. In vitro digestion studies demonstrated that the QPI/SA-stabilized Pickering emulsion with 60% oil phase effectively delayed curcumin release in simulated gastric conditions and facilitated targeted release in intestinal fluids, reaching a release rate of 88.21%. Moreover, cytotoxicity assays indicated that QPI/SA-based emulsions displayed good biocompatibility within a defined concentration range, while curcumin-loaded QPI/SA emulsions exhibited significant inhibitory effects on HeLa cells in a dose-dependent manner. These findings highlight the potential of QPI/SA composite particles as promising food-grade emulsifiers for the development of stable Pickering emulsions and efficient delivery systems for lipophilic functional ingredients.

## Data Availability

The original contributions presented in this study are included in the article. Further inquiries can be directed to the corresponding authors.
